# Epithelial Barrier Dysfunction in Diarrhea-Predominant Irritable Bowel Syndrome (IBS-D) via Downregulation of Claudin-1

**DOI:** 10.3390/cells12242846

**Published:** 2023-12-15

**Authors:** Karem Awad, Christian Barmeyer, Christian Bojarski, Oliver Nagel, In-Fah M. Lee, Michal R. Schweiger, Jörg-Dieter Schulzke, Roland Bücker

**Affiliations:** 1 Clinical Physiology/Nutritional Medicine, Department of Gastroenterology, Infectious Diseases and Rheumatology, Charité—Universitätsmedizin Berlin, Campus Benjamin Franklin, 12203 Berlin, Germanyoliver.nagel@fu-berlin.de (O.N.);; 2 Institute for Translational Epigenetics, Medical Faculty, University of Cologne, 50931 Cologne, Germany

**Keywords:** irritable bowel syndrome, intestinal barrier function, tight junctions, claudin-1, tricellulin, leaky gut, RNA-seq

## Abstract

Background: In patients with diarrhea-predominant irritable bowel syndrome (IBS-D), the diarrheal mechanisms are largely unknown, and they were examined in this study on colon biopsies. Methods: Electrophysiological measurements were used for monitoring functional changes in the diarrheic colon specimens. In parallel, tight junction protein expression was analyzed by Western blot and confocal laser-scanning microscopy, and signaling pathway analysis was performed using RNA sequencing and bioinformatics. Results: Epithelial resistance was decreased, indicating an epithelial leak flux diarrheal mechanism with a molecular correlate of decreased claudin-1 expression, while induction of active anion secretion and impairment of active sodium absorption via the epithelial sodium channel, ENaC, were not detected. The pathway analysis revealed activation of barrier-affecting cytokines TNF-α, IFN-γ, IL-1β and IL-4. Conclusions: Barrier dysfunction as a result of epithelial tight junction changes plays a role in IBS-D as a pathomechanism inducing a leak flux type of diarrhea.

## 1. Introduction

Diarrhea can arise from intestinal malabsorption, active secretion or leak flux as a result of barrier defects, as well as motility disorders of the intestine [1]. Chronic manifestation can be the result of a substantial number of different diagnoses, including infections such as lambliasis, amoebiasis or strongyloidiasis, as well as AIDS-related infections. Additionally, it can also be a consequence of immune- or autoimmune-related disorders, such as inflammatory bowel diseases (IBD), including microscopic colitis, neuroendocrine etiology, e.g., carcinoid syndrome, gastrinoma, VIPoma, or irritable bowel syndrome (IBS) [2].

IBS is a chronic functional disorder of the gastrointestinal tract with a prevalence of 10–20% [3]. It is characterized by abdominal pain and changes in bowel movements (diarrhea and/or constipation). According to the patient’s reported primary bowel habit, there are four different forms of IBS: IBS-D with predominantly diarrhea, IBS-C with predominantly constipation, IBS-M with mixed bowel habits and unclassified IBS-U [4,5].

While in IBS-C and IBS-M, obstipation is predominant—which is interrupted in IBS-M by solving diarrheal phases—IBS-D is characterized by ongoing diarrheal states. This diagnosis in particular requires the exclusion of somatic diseases by means of laboratory tests, imaging and microbiological testing [6]. Often, this follows acute gastrointestinal infections with bacterial pathogens or *Giardia lamblia* persisting in the long term even after successful antibiotic therapy. Despite being common throughout the world’s population [7], the pathogenesis of IBS-D is still not completely understood. However, the efficacy for a therapeutic strategy will benefit from an increasing understanding of the underlying pathomechanisms.

One aspect of intestinal dysfunction in IBS is an impaired barrier function, for which a central role in pathophysiology is discussed [8]. A previous study showed that microRNA-16 modulates epithelial tight junctions (TJ) via TLR4/NF-κB pathway in IBS-D [9]. Structural changes in cellular TJ proteins have also been described by others to be associated with IBS. Cheng et al. showed a downregulation of claudin-1 in IBS-D patients, while it was increased in IBS-C patients [10]. The expression of claudin-2 was shown to be significantly elevated in the ileum of patients diagnosed with diarrhea-predominant irritable bowel syndrome, while its expression was not altered in the rectal mucosa of patients diagnosed with IBS-D [11].

TJ strands are formed by a multi-protein complex consisting of claudins, junction adhesion molecules (JAMs) and tight-junction-associated MARVEL proteins (TAMPs) [12]. These highly regulated components play a crucial role in the closure of intercellular gaps, effectively controlling the passage of ions and molecules of either size across the epithelium. Furthermore, TJs maintain the epithelial cell polarity by separating the apical and basolateral plasma membrane compartments, restricting the lateral membrane diffusion of membrane proteins (fence function). Occludin, tricellulin and the claudin family of TJ proteins, with 27 distinct claudins identified in mammals [13], are functionally important TJ protein components with either a barrier- or channel-forming activity. The majority of claudins are recognized for their role in tightening cellular junctions, such as claudin-1, claudin-3, claudin-4, claudin-5 and claudin-8. Conversely, claudin-2, claudin 10a and 10b, claudin-12 and claudin-15 are known to operate as ion channels [14,15,16,17,18], which could contribute to diarrhea via a pore pathway, resulting in a leak flux mechanism when overexpressed.

Furthermore, it has been proposed that intestinal barrier dysfunction could be linked to visceral hypersensitivity in IBS patients [19]. The activation of mast cells and the movement of mast cells into the vicinity of epithelial cells or sensory nerves in the intestinal mucosa lead to an interaction between the tissues and the immune cells, which results in barrier dysfunction and visceral hypersensitivity via the release of mediators, such as prostaglandin E2, histamine, tryptase and cytokines [20]. Epithelial barrier disruption is frequently paralleled by hypersensitivity in IBS, worsening abdominal pain in this disorder [20]. A previous clinical study found that IBS-D and post-infectious IBS patients have an increased small intestinal permeability for 339 Da ^51^Cr-EDTA compared with the controls [21]. This brings the phenomena of an acute infectious barrier impairment and the prolongation of this barrier defect along the *leaky gut* concept together with the onset of IBS.

However, it has now also become clear that microbial factors are very important in the biology of IBS. This includes differences in the microbiota in the two main types of IBS, namely IBS-D and IBS-C. IBS-D showed an overgrowth of small intestinal bacteria, which can be identified by a positive hydrogen breath test, while IBS-C had higher levels of methanogenic archaea [22].

However, little is now known so far about the IBS-D subgroup’s functional changes in the large intestinal mucosa responsible for diarrhea. In particular, no direct measurements were performed in humans regarding the intestinal barrier function. Furthermore, the pattern of TJ protein expression in IBS-D has not yet been fully characterized. In order to better characterize IBS-D, we examined (i) ion permeability, as measured with impedance spectroscopy, (ii) paracellular macromolecule permeability in colonic biopsies, (iii) the expression pattern of TJ proteins and (iv) the subcellular localization of barrier-relevant TJ proteins. In addition, RNA sequencing (RNA-seq) using bioinformatics pathway analysis was conducted to clarify the signaling pathways, which are implicated in IBS-D.

## 2. Materials and Methods

### 2.1. Study Design

We included seven patients with IBS-D (four female, three male) and ten controls (five female, five male) in the observation study. In order to diagnose IBS-D, the Rome III criteria were used. The criteria include abdominal pain or discomfort, which has occurred at least three days per month for the past three months and is linked to two or more criteria: (i) improvement with defecation, (ii) onset associated with a change in stool frequency or (iii) onset associated with a change in stool form.

None of the IBS-D patients were taking any medication or showed any intestinal lesions. The average age was 35 ± 4 years (controls: 48 ± 3 years). A colonoscopy with biopsy sampling was conducted for each patient and was also performed on control subjects, for example for prevention of colon cancer. All biopsies were taken 25–35 cm ab ano. The sigmoid colon biopsies were placed into miniaturized Ussing chambers (exposed tissue area: 0.049 cm^2^) for functional measurements, and in parallel, biopsies were fixed for molecular analysis. It took approximately 30 min from the time the biopsy was taken until it was mounted in the Ussing chamber.

### 2.2. Ethics

This study complied with the Helsinki Declaration regarding the use of human subjects. Each patient provided written informed consent for the study, which was authorized by the institutional review board of “The Ethics Committee of the Charité” under the approval number EA4/015/13.

### 2.3. Ussing Chamber Measurements

The colon biopsies were affixed into miniaturized Ussing chambers, which had an area of 0.049 cm^2^. The colon mucosa’s total transepithelial electrical resistance (R_t_ = TER) comprises two distinct components: the epithelial resistance (R_epi_) and the subepithelial resistance (R_sub_). These components can be distinguished by using impedance spectroscopy [23]. The experimental setup involved the utilization of a programmed frequency response analyzer (Model 402, Beran Instruments, Devon, UK) and an electrochemical interface (Model 1286, Solartron Schlumberger, Farnborough, UK) to apply alternating currents within the frequency range of 1 Hz to 65 kHz. The impedance was derived from the voltage responses. The electrical resistance of the bath was used for correction. The complex impedance values were presented in a Nyquist diagram subsequent to the adjustment of the resistance of the bathing solution and the frequency response of the measuring equipment at each frequency.

The total transepithelial resistance R_t_ was determined by identifying the point of intersection between the semicircle and the *x*-axis at a frequency of 0. Similarly, the R_sub_ was determined by identifying the point of intersection between the semicircle and the *x*-axis at an infinitely high frequency. The R_epi_ was calculated by subtracting the R_sub_ from the R_t_.

The bathing solution for Ussing chamber studies and the atmospheric conditions were the same as in a similar study described previously [24].

Tissue vitality was assessed subsequent to each Ussing experiment. In order to achieve the intended objective, the activation of electrogenic chloride secretion was stimulated by prostaglandin E2 (PGE2, 10^−6^ mol/L), theophylline (10^−2^ mol/L) and carbachol (10^−4^ mol/L) (all Sigma-Aldrich, St. Louis, MO, USA).

### 2.4. Electrogenic Sodium Absorption

Aldosterone (3×10^−9^ mol/L, Sigma-Aldrich, St. Louis, MO, USA) was administered to human biopsies during the Ussing chamber experiments to simulate steroid stimulation as present in diarrheal conditions. The activity of the epithelial sodium channel (ENaC) was assessed eight hours following stimulation. This was accomplished by introducing the ENaC blocker amiloride (10^−4^ mol/L, Sigma-Aldrich, St. Louis, MO, USA) into the mucosal compartment. The subsequent decrease in short-circuit current (I_SC_) was attributed to ENaC-dependent sodium absorption, as previously documented [25].

### 2.5. Electrogenic Anion Secretion

The I_SC_ of human colon biopsies was measured in parallel Ussing experiments in order to quantify basal rheogenic anion secretion. At the end of the experiment, bumetanide (Sigma-Aldrich, St. Louis, MO, USA) was added with a concentration of 10^−5^ mol/L to the serosal side in order to quantify electrogenic chloride secretion as the bumetanide-sensitive I_SC_. 

### 2.6. Permeability Analysis

The functional measurement of macromolecule permeability was performed as described previously [24]. Briefly, tracer flux measurements conducted under voltage-clamped conditions in Ussing chambers were used to determine the permeability for the paracellular flux marker fluorescein. A volume of 100 µmol/L fluorescein (Sigma-Aldrich, Darmstadt, Germany) was added to the apical side. The basolateral samples were obtained at time intervals of 15, 30, 45 and 60 min.

In parallel experiments, 0.4 mmol/L FITC-dextran 4 kDa was applied apically, while unlabeled 4 kDa dextran was added on the basal side of the tissue (both Sigma-Aldrich, Darmstadt, Germany). Fluorescence analysis was conducted in duplicate using a spectrophotometer plate reader at a wavelength of 535 nm (Tecan Infinite M200, Tecan, Männedorf, Switzerland). The fluorescence measurements were calibrated using predetermined dilutions. The permeability of fluorescein or FITC-dextran was determined using the formula P = J/Δc, with P = permeability (cm/s), J = flux (mol∙h^−1^∙cm^−2^) and c = concentration (mol/L).

### 2.7. Protein Expression

Colonic samples were subjected to Western blot analysis. Protein extraction and homogenization were performed by utilizing a lysis solution containing the following components: 20 mmol/L Tris (pH 7.4), 5 mmol/L MgCl_2_, 1 mmol/L EDTA, 0.3 mmol/L EGTA and a complete protease inhibitor combination (Roche AG, Basel, Switzerland). The extraction process involved passing the mixture through a 27.5 G needle. The insoluble material was removed from the extract by centrifuging it at 200× *g* for 5 min at 4 °C, and then, the supernatant was centrifuged at 43,000× *g* for 30 min at 4 °C.

Western blot analysis was performed as described previously [24]. Briefly, the pellet (membrane fraction) was reconstituted in a lysate buffer. The process of separating 20 µg of protein was carried out using polyacrylamide gel electrophoresis, followed by transferring the separated proteins onto a PDVF membrane (Perkin Elmer, located in Rodgau, Germany). The generation of blots involved the use of primary rabbit (rb) polyclonal IgG antibodies against claudin-1, -2, -3, -4, -5, -8 and occludin (rb claudin-1 #519000, rb claudin-2 #516100, rb claudin-3 #341700, m claudin-4 #329400, rb claudin-5 #341600, rb claudin-8 #710222, rb occludin #711500; all Invitrogen, Karlsruhe, Germany) and the primary mouse monoclonal IgG antibodies against β-actin (Sigma-Aldrich, St. Louis, MO, USA).

The quantification of protein expression was performed using densitometry with luminous imaging (LAS-1000, Fuji Film, Tokyo, Japan). The AIDA software (Raytest, version 3.2.1, Berlin, Germany) was used for data analysis.

### 2.8. Analysis of Subcellular Tight Junction Distribution

Immunofluorescence microscopy is a technique for accurate visualization of the structures within tissues and cells by using antibodies. In this work, immunofluorescence was used to examine the subcellular localization of TJ proteins in the sigmoid colon, as described previously [24].

Briefly, sigmoid biopsies were subjected to fixation in a 1% paraformaldehyde solution for a duration of 60 min at room temperature. Subsequently, the samples were subjected to washing in PBS+Ca/Mg solution, followed by incubation in a 25 mmol/L glycine solution for a duration of five minutes. Following a second round of washing in PBS+Ca/Mg, the biopsies underwent a sequential dehydration process using an increasing sucrose series (10%, 20% and 30%) at room temperature for 60 min. In order to produce the cryoblocks, the biopsies underwent a freezing process using methylbutane frozen with liquid nitrogen. Following this, the biopsies were placed in TissueTek (Sakura Finetek, Europe B.V, Umkirch, Germany) and stored at a temperature of −80 °C. A cryostat (Leica CM 1950, Leica, Wetzlar, Germany) was used to prepare tissue sections of 5 μm thickness. Before they were stained, these were frozen on coated slides at −20 °C for 12–24 h. Permeabilization was achieved by adding 0.5% Triton-X to the tissue sections for 10 min. Subsequently, unspecific binding was prevented with a blocking solution for 60 min (1% Goat serum, 5% BSA, 0.05% Triton X-100).

Finally, the main antibody (tricellulin #700191, 1:100, Invitrogen, Karlsruhe, Germany) was added overnight at 4 °C. There were three washing steps after the first incubation step. Then, the fluorescent secondary antibodies Alexa Fluor-488 goat anti-rabbit (#A32731) and Alexa Fluor-594 goat anti-mouse (#A32742, 1:500, Invitrogen, Karlsruhe, Germany) were used for 120 min at 37 °C. DAPI (4′-d-diamidino-2-phenylidole dihydrochloride, 1:1000, Roche AG, Mannheim, Germany) was used to stain the nuclei. Afterward, Pro Tags Mount Fluor (Biocyc, Potsdam, Germany) was used to mount them on glass slides.

The subcellular distribution of the TJ proteins was determined by using confocal laser-scanning microscopy (Zeiss LSM 780, Zeiss, Jena, Germany) on fluorescence-labeled tissue sections. The secondary antibodies were utilized at excitation wavelengths of 594 nm (red), 488 nm (green) and 358 nm (blue for DAPI). The images were acquired with plan apochromat plan neofluor objectives with numerical apertures of 1.4 at magnifications of 40× and 63×. In order to visualize the primary localization of tricellulin, we performed an intensity plot analysis using the ZEN blue 2.5 lite software (Zeiss LSM ZEN software, version 2.5.75.0, Carl Zeiss Microscopy GmbH, Jena, Germany).

The measurement of tricellulin intensity was conducted in the tricellular tight junction (tTJ) and compared with the intensity of tricellulin in the bicellular tight junction (bTJ) located 2 µm away from the measurement location of three cells in the tTJ. The measurements were performed on 3 controls in comparison to 3 patients with IBS-D. Measurements were taken 3 times in each of the 3–6 sections for each patient.

### 2.9. Morphometry of the Mucosal Surface Area

The calculation of mucosal surface area was performed in both individuals with IBS-D and control subjects, as the transport and barrier characteristics are known to be influenced by the mucosal architecture.

In this study, the tissues were promptly fixed with 4% formalin immediately following the Ussing experiments. Subsequently, the tissues were embedded in paraffin using the same degree of stretch as in the electrophysiological experiments.

H&E sections (hematoxylin and eosin) were prepared and examined with light microscopy in low-power fields (10× magnification). Image J was used to perform morphometry on digitally stored light micrographs. The inner crypt diameter in cross-sections of the tissues, crypt length and crypt density were all measured by morphometry.

### 2.10. TUNEL Assay

Apoptotic cells can be specifically stained by the terminal transferase dUTP nick end labeling (TUNEL) assay. Thew staining for apoptosis was performed as described previously. Briefly, biopsies fixed in 4% paraformaldehyde underwent morphological assessments. Paraffin-embedded specimens were serially sectioned. Terminal deoxynucleotidyl transferase-mediated deoxyuridine triphosphate nick-end labeling (TUNEL, In Situ Cell Death Detection Kit- Fluorescein, Roche, Mannheim, Germany) was used to label DNA fragments.

Highly condensed, highly fluorescent and multi-segmented nuclei were considered apoptotic. The apoptosis rate is equal to the percentage of apoptotic epithelial cells out of all epithelial cells visible in the field of view (~150 epithelial cells/field of view). The apoptosis rate was determined for each patient as a mean value from at least five different visual fields.

### 2.11. RNA Expression Analysis

We analyzed differential gene expression using RNA-seq followed by bioinformatics-based pathway analysis, as described previously [24]. Biopsies of the sigmoid colon were processed with the mirVana RNA isolation reagent (Ambion, Life Technologies, Carlsbad, CA, USA) to isolate total RNA. RNA sequencing was performed using the TrueSeq Stranded Total RNA technique on an Illumina NovaSeq 6000 sequencing system with RNA quality values of 80%. The RNA sequences acquired from RNA-seq were aligned with the human genome GRCh38 release 97 and organized using the STAR aligner version 2.7.1a in a two-pass mode. The frames for first-pass read mapping were determined using the coordinates from Ensembl Annotation Release 97. The gene-count tables, which include gene-read coverages, were generated using the Counts Function of the Bioconductor package Rsubread. The Bioconductor package DESeq2 was applied to assess the differential expression of genes between two states, quantifying log2-fold changes and their respective *p*-values. The pathway analysis and upstream regulator analyses were conducted using the Ingenuity Pathway Analysis program (IPA, Qiagen Silicon Valley, Redwood, CA, USA).

The raw unprocessed sequencing data, in the form of Fastq files, and a raw data matrix table were deposited in the European Genome-Phenome Archive (EGA) database under record number EGAD50000000063 (https://ega-archive.org/datasets/EGAD50000000063, accessed on 10 November 2021).

### 2.12. Statistics

For statistical analysis, the GraphPad Prism software version 10.0 (GraphPad Software Inc., San Diego, CA, USA) was used. Data are expressed as mean value and standard error of the mean (SEM). The *p*-values were calculated using Student’s *t*-test. *p* < 0.05 was considered significant.

## 3. Results

### 3.1. Active Chloride Secretion Is Not Altered in the Colon of IBS-D Patients

**Figure 1:**
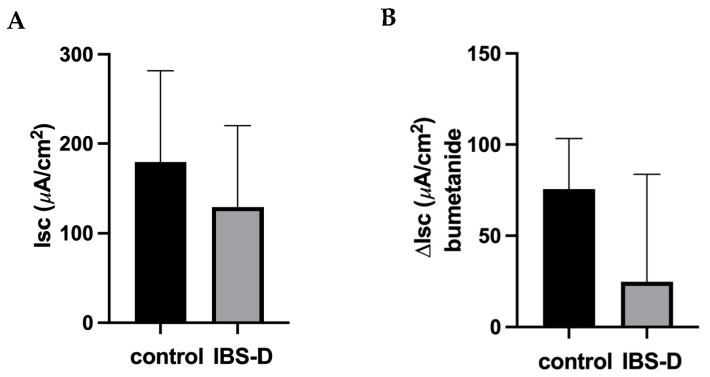
Active electrogenic chloride secretion in IBS-D patients. Short-circuit current (I_SC_, in µA·cm^−2^) of sigmoid colon mucosa from controls and patients with diarrhea-predominant irritable bowel syndrome (IBS-D) was measured. (**A**) Baseline I_SC_ was determined in controls (*n* = 9) and IBS-D patients (*n* = 7). (**B**) Bumetanide was used to identify active electrogenic chloride secretion by inhibiting the Na-K-Cl co-transporter (NKCC1). The difference between the maximum I_SC_ before and 20 min after adding bumetanide to the basolateral side of the Ussing chamber is given as ΔI_SC_ bumetanide. The individual short-circuit current values were corrected using the ratio of total electric wall resistance over epithelial resistance (formula R_t_/R_epi_; 1.33 ± 0.08 for controls versus 1.41 ± 0.05 for IBS-D; n.s.). Data represent mean ± SEM.

In Ussing chambers, the transport and barrier functions of mucosal biopsies from the distal colon were investigated in order to identify diarrheal mechanisms. The baseline short-circuit current (I_SC_) indicates active electrogenic transport activity (Figure 1). The source of this I_SC_ in the large intestine under baseline conditions is active anion secretion, i.e., active secretion of chloride and/or bicarbonate [26]. However, there was no difference in electrogenic transport between IBS-D patients and controls (Figure 1A). In addition, active electrogenic chloride secretion was specifically quantified as bumetanide-sensitive I_SC_ but turned out to be unaltered as well (Figure 1B). Accordingly, RNA sequencing (RNA-seq) did not show altered mRNA expression of transporters involved in active chloride secretion, such as NKCC1, CLCA1 and CFTR (Table 1).

### 3.2. Unaltered Epithelial Sodium Channel Transport Function in IBS-D

We studied the activity of the epithelial sodium channel (ENaC)—the rate-limiting transport protein of active sodium uptake in the distal colon and rectum—because sodium malabsorption is a possible diarrheal mechanism. The biopsy samples from IBS-D patients and controls were analyzed for 6–8 h in Ussing chambers under stimulation with 3 nmol/L aldosterone, mimicking the stimulation situation in a diarrheal state.

**Figure 2:**
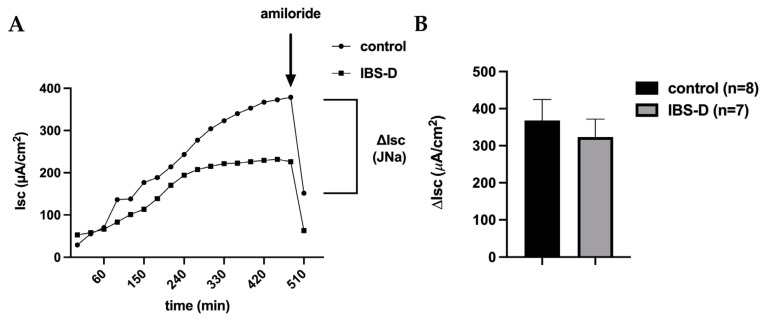
Electrophysiological measurements of active sodium absorption via the epithelial sodium channel (ENaC) in the colon mucosa of IBS-D patients and controls. (**A**) Representative curves of ENaC activity measurements. Electrogenic sodium absorption (J_Na_) was obtained as a decrease in short-circuit current (ΔI_SC_) following the addition of amiloride, a specific ENaC channel blocker, under stimulation with aldosterone. The squares show values from an IBS-D patient, and the circles show values from a healthy control, representing data from two separate experiments. (**B**) Colon biopsies of 8 controls and 7 patients with IBS-D were studied for ENaC-dependent electrogenic sodium transport in miniaturized Ussing chambers. Data represent mean ± SEM. The values were corrected for subepithelial resistance by R_t_/R_epi_.

The quantification of electrogenic sodium absorption (J_Na_) was achieved by measuring the decrease in I_SC_ following the addition of 10^−4^ mol/L amiloride (Figure 2A). The results are presented as means ± SEM of eight controls and seven IBS-D patients, respectively. Nevertheless, there was no difference when comparing the control group to those diagnosed with IBS-D, as depicted in Figure 2B. Thus, ENaC-dependent active sodium absorption in the distal colon was unaffected in IBS-D patients.

In accordance with the functional observation shown in Figure 2B, our analysis of RNA-seq data (Table 2) obtained from the colonic mucosae of individuals with IBS-D and the controls did not show any alterations in gene expression levels of the ENaC subunits α, β and γ (SCNN1A, SCNN1B, SCNN1G; n.s.). The sequencing data were deposited under the accession number EGAD50000000063.

### 3.3. Reduced Ion Permeability in IBS-D Patients in Impedance Spectroscopy

**Figure 3:**
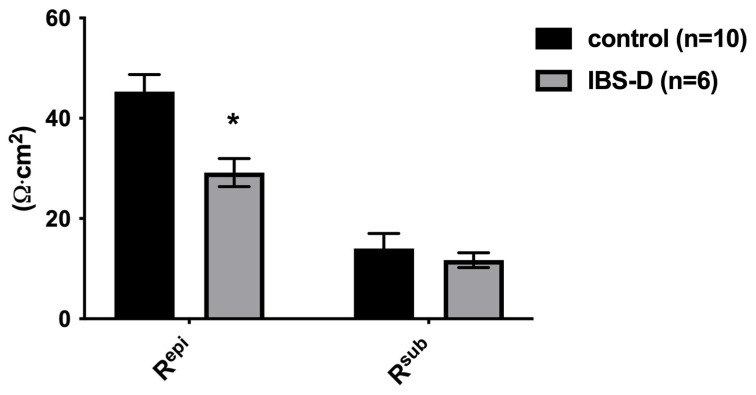
Epithelial and subepithelial resistances in IBS-D and controls. The total wall resistance (R_t_), the epithelial resistance (R_epi_) and the subepithelial resistance (R_sub_) were analyzed by impedance spectroscopy in endoscopically obtained colon biopsies in miniaturized Ussing chambers. R_epi_ was reduced in patients with IBS-D. Controls: *n* = 10; IBS-D: *n* = 6, * *p* < 0.05. R_sub_ was not altered in comparison with controls. The correction factor R_t_/R_epi_ of I_SC_ values for R_sub_ contributions in the Ussing chamber did not differ from controls (R_t_/R_epi_: 1.33 ± 0.08 for controls versus 1.41 ± 0.05 for IBS-D; n.s. = not significantly different from controls). Statistical comparison was performed using Student’s *t*-test. Data represent mean ± SEM.

Distinguishing the epithelial (R_epi_) and subepithelial (R_sub_) resistance proportions of the total intestinal wall resistance (R_t_), as exposed in the Ussing chamber with impedance spectroscopy, revealed that IBS-D patients have a R_epi_, which is reduced to 64% of that of controls (29 ± 3 Ω∙cm^2^ (*n* = 6) versus 45 ± 3 Ω∙cm^2^ (*n* = 10) in controls, * *p* < 0.05; Figure 3). In contrast, R_sub_ did not differ between both groups (Figure 3 legend), which corresponds to the subepithelial tissue layers appearing unchanged in macroscopic and microscopic analyses, since microscopic signs of inflammation were absent in IBS-D (see also Section 3.9).

### 3.4. Unaltered Macromolecule Permeability in IBS-D Patients

**Figure 4:**
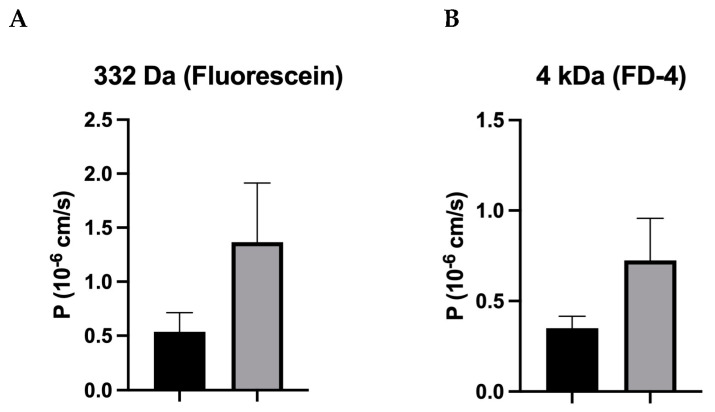
Permeability for macromolecules in IBS-D. (**A**) In IBS-D, 332 Da fluorescein did not show altered permeability compared with controls. Controls: *n* = 10; IBS-D: *n* = 6; n.s. (**B**) The permeability for FITC-dextran-4000 (4 kDa) in IBS-D patients was also not altered compared with controls. Controls: *n* = 8; IBS-D: *n* = 6; n.s. Data represent mean ± SEM.

In contrast to ion permeability, macromolecular tracer flux measurements revealed no alteration in permeability for 332 Da fluorescein (Figure 4A) and for 4 kDa FITC-dextran in IBS-D colon biopsies (Figure 4B). However, a tendency toward increased macromolecular permeability is recognizable, but this does not reach statistical significance.

### 3.5. Tight Junction Protein Expression: Claudin-1 Is Reduced in IBS-D

**Figure 5:**
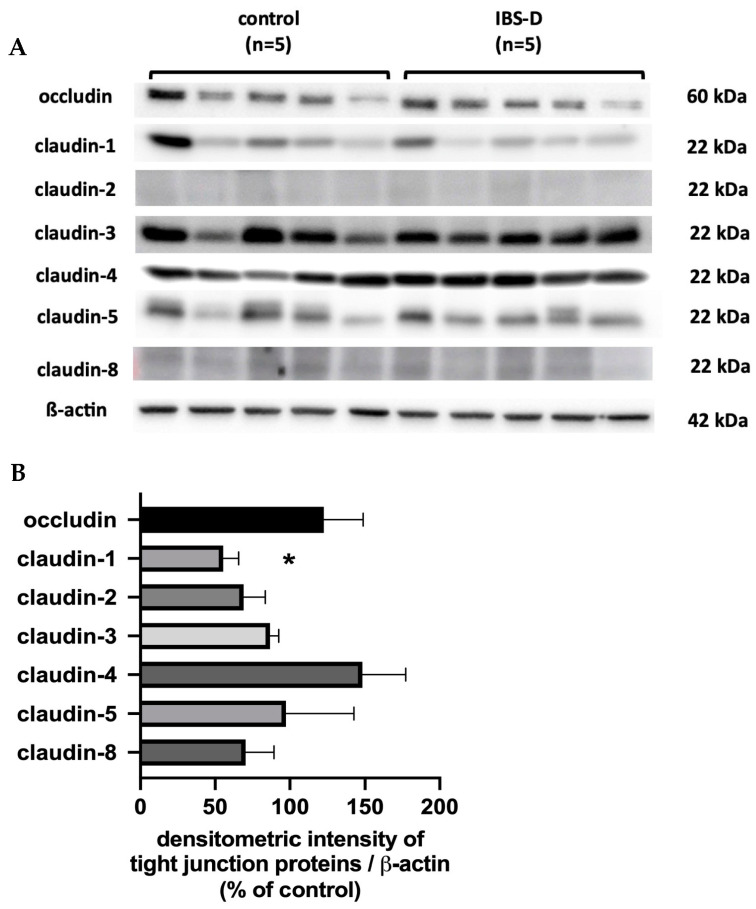
Western blots and densitometry results of strand-forming TJ proteins in IBS-D. (**A**) Representative Western blots are shown for occludin, claudin -1, -2, -3, -4, -5 and -8. (**B**) Densitometry results from Western blots of controls and patients with IBS-D (*n* = 5–10). The expression level of proteins from control subjects was set to 100%. * *p* < 0.05 when comparing controls and IBS-D in Student’s *t*-test. Data represent mean ± SEM.

To determine the structural correlate of the reduced epithelial resistance in IBS-D, we examined the Western blot expression of strand-forming TJ proteins. Occludin, claudin-1, claudin-2, claudin-3, claudin-4, claudin-5, claudin-8 and tricellulin were examined. However, the majority of TJ proteins were not altered in IBS-D. Representative Western blots are shown in Figure 5A, and their densitometry is given in Figure 5B. Most importantly, the expression of claudin-1 was decreased in IBS-D.

### 3.6. Tricellulin Is Not Redistributed off the Tricellular Tight Junction in IBS-D

**Figure 6:**
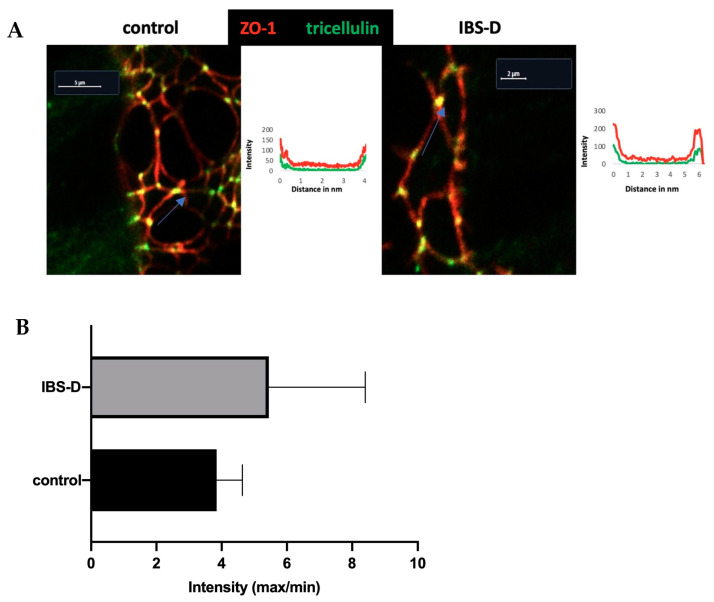
Immunofluorescence detection of TJ proteins in IBS-D patients with confocal laser-scanning microscopy (CLSM). (**A**) Crypt regions from a control and an IBS-D colon biopsy. The co-localization of tricellulin (green) and ZO-1 (red) appears yellow (merge). Tricellulin staining in the TJ did not appear attenuated in IBS-D compared to controls. Furthermore, there is no evidence that tricellulin was sorted out of the tricellular tight junction (tTJ). Distance–intensity plots show where tricellulin is located in tTJs. As indicated by the blue arrow in the control specimen (marked as the length and the direction of the pixel intensity measurement along the TJ), tricellulin (green) exhibits distinct signal maxima at tTJ. In IBS-D, the localization of tricellulin was not altered compared to controls. (**B**) The intensity of tricellulin was measured in the tTJ in relation to the intensity in the bicellular tight junction (bTJ) at a distance of 2 µm from the measuring point in the tTJ. In comparison to controls, the intensity ratio of tricellulin (tTJ/bTJ) was not altered in IBS-D (*n* = 3 in each group; n.s.).

In addition to the quantification of TJ proteins in Western blots, we determined the subcellular localization of strand-forming TJ proteins in order to determine whether the loss of TJ proteins from TJ strands contributed to the epithelial barrier impairment. Sections of immunostained colon tissue were examined using confocal laser-scanning microscopy (CLSM). However, tricellulin localization was not significantly altered in IBS-D patients (Figure 6A). This is supported by tricellulin immunofluorescence intensity plots comparing tricellular and bicellular localization (Figure 6B).

### 3.7. RNA Sequencing Analysis in IBS-D Patients

In order to investigate the potential impact of mRNA alterations on epithelial transport and barrier function in the colon of IBS-D patients, an analysis of RNA-seq data obtained from colon biopsies was performed. The expression of occludin, claudin-1, claudin-3, claudin-4, claudin-5 and claudin-8 mRNA was evaluated. The analysis showed an altered expression of the mRNA only for claudin-4 (Table 3), which was increased in IBS-D. The RNA-seq data showed no reads for claudin-2, either in IBS-D or in controls, which is not surprising, however, since claudin-2 is usually upregulated only in epithelial inflammation or restitution states. The fact that the decrease in protein expression of claudin-1 was not paralleled by a change in claudin-1 mRNA level indicates post-transcriptional regulation and/or accelerated protein degradation of claudin-1 in IBS-D patients.

### 3.8. Pathway Analysis of IBS-D

The RNA-seq results were used for analysis with the bioinformatics pathway analysis tool Ingenuity Pathway Analysis (IPA) software (version 101138820). This software utilizes the mRNA expression pattern of genes connected to specific pathways in order to predict the activation or inhibition of signaling pathways (e.g., via Upstream regulator analysis). The results of this analysis suggest that cytokines and lipopolysaccharides (LPS), as presented in Table 4, play a significant role in the pathogenesis of IBS-D. These molecules exhibit potent effects on downstream target genes, indicating their elevated signaling activity in this condition (Table 4, Supplementary Table S1). This observation suggests that the involvement of a mucosal immune response has a role in the functional impairments observed in IBS-D. In addition to the signaling in the immune function in Table 4, the IPA analysis of the mucosa specimens from IBS-D patients revealed alterations in other pathways, e.g., in the testosterone cycle. Notably, dihydrotestosterone (DHT) (with a z-score of 2.26 and a *p*-value of overlap of 1.64∙10^−3^) was identified as a hint of an involvement of sex hormone pathways (Supplementary Table S1). Additional signaling pathways associated with micronutrients, such as calcitriol and tretinoin, were also shown to be activated (the z-score for calcitriol was 2.21, with a *p*-value of overlap equal to 1.03∙10^−2^; the z-score for tretinoin was 3.37, with a *p*-value of overlap equal to 5.45∙10^−4^; Supplementary Table S1). Both substances showed immunomodulatory pharmaceutic properties in the gut. This implies that the intake of these compounds may lead to favorable regulatory effects. Nevertheless, it is important to point out that bioinformatics predictions of this kind offer only preliminary findings, and actual confirmation, as shown in the current study, is required in relation to barrier function.

### 3.9. Mucosal Architecture and Epithelial Apoptotic Rate Remained Unchanged in IBS-D

**Figure 7:**
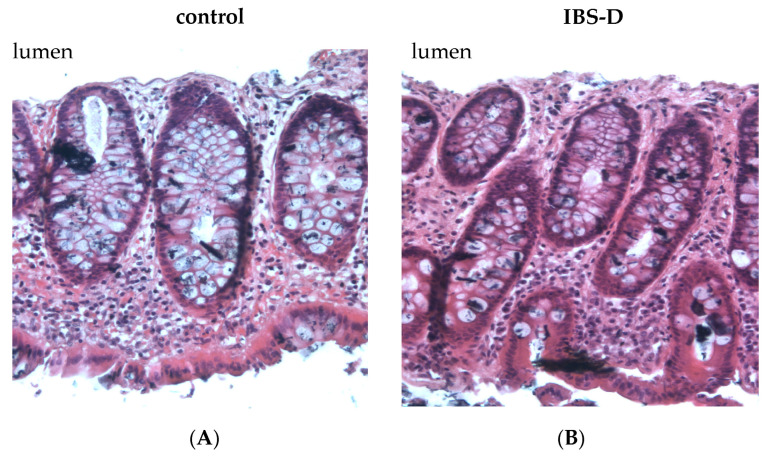
Histological analysis of the sigmoid colon of patients with IBS-D. Representative biopsy specimens from the sigmoid colon of (**A**) controls and (**B**) IBS-D patients were stained with H&E and visualized using a microscope with 20-fold magnification. There were no gross lesions or surface area differences between healthy controls and IBS-D.

The impairment of epithelial TJs is commonly associated with alterations in epithelial resistance/conductance and transepithelial tracer fluxes. However, it is important to note that changes in the mucosal architecture, such as an increase in crypt length, can increase the exposed TJ area within the Ussing chamber. This can also contribute to resistance or tracer flux alterations without any TJ protein modification. Moreover, an elevation in the rate of epithelial apoptosis or the presence of epithelial gross lesions can affect the integrity of the barrier. However, our conventional histology did not reveal any erosions or ulcers in the IBS-D mucosae (Figure 7). Morphometric analysis of both mucosae also revealed no difference in the surface area between IBS-D patients and controls (Table 5 corresponding to Figure 7). In the TUNEL-stained tissue sections, there was also no significant difference observed in the epithelial apoptotic rate between controls and IBS-D patients (*n* = 5 for each group; n.s.; Table 5).

## 4. Discussion

Given the high heterogeneity of patients with IBS, we aimed for the most precise definition possible for our study group. Therefore, we specifically included only individuals diagnosed with IBS-D. As one of the main unsolved questions in IBS research, the pathogenesis of diarrhea in the IBS-D subgroup is not clearly understood. Therefore, in Part A of our study, we analyzed colon biopsies from IBS-D patients maintained functionally intact under in vitro conditions to distinguish the three main mechanisms of diarrhea, namely malabsorption, induction of active secretion and leak flux diarrhea secondary to barrier dysfunction. Thus, this is the first study, which carried out a systematic investigation of all possible mechanisms of diarrhea in the colonic mucosa and analyzed the expression of the strand-forming TJ proteins expressed in the colon.

The first important result we found was evidence of a defect in the epithelial barrier in IBS-D, namely a reduced epithelial resistance in impedance spectroscopy. This reduction in epithelial resistance could be due to various factors, which may occur independently or in combination. These include changes in the structure of the TJs, in the epithelial apoptosis rate, altered mucosal architecture with altered mucosal surface geometry and the appearance of mucosal gross lesions, such as erosions. To determine the characteristics of the epithelial barrier defect in IBS-D, in Part B, we conducted a comprehensive experimental study on the structural aspects of the epithelial barrier in the large intestine.

According to our results, diarrhea in IBS-D is not explained by the activation of active anion/chloride secretion, as neither the basal I_SC_ in IBS-D biopsy specimens nor the bumetanide-sensitive I_SC_ were altered. In support of this view, in our accompanying mRNA-seq analysis of these IBS-D biopsy samples, the expression of components of the active chloride secretion system (such as NKCC1, CLCA1 and CFTR) was not altered. In the past, we also checked the activation of chloride secretion in other groups of patients with different diseases using exactly the same measuring system in the same intestinal segment—the sigmoid colon. In another cohort of IBS-M patients, chloride secretion was found to be even reduced, consistent with the constipation phase of these patients [24]. In contrast, we found that chloride secretion was activated in patients with collagenous colitis and diarrhea [23], proving that our measuring system is capable of detecting active anion secretion states through I_SC_ measurements in miniaturized Ussing devices.

In addition to active ion secretion, diarrhea in IBS-D could also be due to a malabsorptive mechanism. In the distal colon and rectum, active sodium absorption via the ENaC is the main absorptive transport system. In one of our previous studies, we demonstrated that ENaC-dependent sodium absorption is significantly impaired in lymphocytic colitis [25]. Therefore, we also tested this absorptive transport system in IBS-D. However, unlike in lymphocytic colitis, it was not reduced in IBS-D compared to controls. Unimpaired ENaC function in IBS-D is another important finding of our study, as impairment may promote diarrhea as a relevant pathomechanism in the large intestine, as it has been shown to be involved in patients with ulcerative colitis [27] or campylobacteriosis [28].

Since it turned out that neither the activation of active secretion nor malabsorption were involved in the diarrhea in IBS-D, a disturbed barrier function for electrolytes and water must be assumed to be the only and predominant pathomechanism of diarrhea. To investigate the structures responsible for this barrier dysfunction, we took a closer look at the epithelial TJs in IBS-D. For this purpose, strand-forming TJ proteins were examined. Based on the functional and expression data from the sigmoid colon, claudins -1, -2, -3, -4, -5 and -8, as well as occludin and tricellulin, are considered the key components of the TJ-determining epithelial barrier function in the colon. In our analysis, we found that only claudin-1 was decreased in IBS-D, while the other strand-forming claudins examined were not altered in their expression. However, the reduction in the barrier-forming TJ protein claudin-1 is certainly suitable to explain the decrease in epithelial barrier function in our analysis.

Martínez and co-workers, focusing on barrier properties in the small intestine, demonstrated increased protein expression of claudin-2, decreased occludin phosphorylation status and increased redistribution of occludin from the plasma membrane to the cytoplasm of enterocytes in IBS-D patients [29]. Taken together, our results and the data of others seem to support the fundamental importance of claudin-1 in the pathogenesis of diarrhea in IBS-D.

To identify the possible regulatory influences on claudin-1 expression, claudin-1 mRNA levels were examined. However, an unchanged mRNA expression level rather suggests post-transcriptional mechanisms of regulation. A prominent candidate for this is increased expression of miRNAs targeting CLDN1, as identified by Zhou and co-workers for miRNA-29 [30], or accelerated degradation through activation of proteases. The latter regulatory mechanism was proposed for occludin by Coëffier and co-workers, who showed increased proteasome-mediated degradation in IBS patients [31].

The recognition and description of subtype-related pathological changes in IBS will make it possible in the future to individualize effective therapies depending on the subtype. For example, glutamine has been described to have a stimulatory effect on claudin-1 expression in the colon mucosa of patients with IBS-D [32].

Contrary to the result of our recent study on IBS-M, which found that tricellulin was redistributed out of the tTJ as a structural correlate of the increased macromolecule permeability [26], this was not observed in IBS-D. Furthermore, in our present study on IBS-D patients, the permeability of macromolecules only tended to be increased and did not reach statistical significance. The fact that there was only one trend without significance was mainly due to the large scattering of the 4 kDa dextran permeability data, which may be due to the heterogeneity in this IBS-D group. This means that at least some of the IBS-D patients could have increased paracellular macromolecular permeability and that other mechanisms of antigen uptake, such as transcytosis, must be assumed for the other patients (see below).

At least in our RNA-seq, we obtained evidence of persistent low-grade immune activation in the IBS-D mucosa. This means that an activating influence on the mucosal immune system can be assumed, which supports the assumption of a resulting impaired epithelial barrier in IBS-D patients, even if this activating influence is only weak and has a rather long-term functional effect. The trigger for such immune activation could be endotoxins, in particular lipopolysaccharides or lipo-oligosaccharides (LPS or LOS) [28]. If this sub-inflammatory immune activation weakens the barrier integrity of the mucosa, this would lead to a vicious cycle of barrier loss and subepithelial immune activation (along the *leaky gut* concept). This interpretation is also supported by the fact that the cytokine profile determined by Upstream regulator analysis in IBS-D included barrier-impairing cytokines and was similar to the cytokine profile previously observed in IBS-M [24], in which a macromolecule permeability increase was measurable.

A particularly noteworthy finding in our present IBS-D study was increased claudin-4 mRNA level, which could be interpreted as a counter-regulatory signal and response to the downregulation of claudin-1 with the aim of maintaining barrier integrity. However, the role of claudin-4 in the pathogenesis of IBS-D is still unclear and requires further investigation. The identification of individual cytokines responsible for barrier dysfunction in IBS-D will enable new therapeutic options. In this way, Huang and co-workers were able to identify a herbal mixture called “QingHuaZhiXie”, which suppressed inflammatory factors and TLR4/MyD88/NF-κB pathway proteins, ultimately leading to increased protein levels of occludin and claudin-1 [33].

In addition to the cytokines (TNF-α, IFN-γ) and LPS in IBS-D, which also play a dominant role in IBS-M, we found in our IPA analysis of RNA-seq data of IBS-D patients that IL-4 is also an upstream regulator. Interestingly, in this context, a recent study from our laboratory found increased transcytotic macromolecule uptake (measured as temperature-dependent horseradish peroxidase flux (HRP, 44 kDa) at 37 °C) in patients with post-infectious irritable bowel syndrome (PI-IBS), resulting in an immune activation [34]. This transcytotic uptake was primarily IL-4-mediated [34]. As already mentioned above, a *leaky gut* component could also be observed in IBS-D, but only in some of the patients, associated with an increased macromolecule permeability to 4 kDa dextran. It is quite possible that in another subset of our IBS-D patients, epithelial transcytosis of antigens could have a functional significance instead. Moreover, the cytokine profiles found here—with cytokines, which influence the epithelium toward the *leaky gut,* such as IL-4 or IL-13, which are similarly activated in infections such as *Campylobacter jejuni* [28]—may also lead to low-grade inflammatory smoldering in IBS as a consequence of previous infectious diarrheal episodes. However, these hypotheses have yet to be confirmed experimentally.

A critical limitation of our present work is that the biopsy samples were collected after an overnight fasting period. Therefore, we cannot exclude with certainty that the stimulation effects from foods, to which IBS patients may react more sensitive than control subjects, have already subsided and were therefore no longer detectable in our Ussing chamber experiment. Thus, we cannot rule out that such stimulatory influence on intestinal secretion is of importance in this context. To exclude this possibility with certainty, transport measurements with a very complex in vivo perfusion system would have to be carried out in these patients.

## 5. Conclusions

In this study, we investigated the mechanisms of diarrhea in IBS-D. One of the most important findings is that the epithelial resistance of the colon was reduced, and this was paralleled by the downregulation of claudin-1. Chloride secretion, ENaC activity or apoptosis induction did not appear to be involved in the pathogenesis of IBS-D. Given the clear evidence of low-grade inflammation of the colonic epithelia, further studies on the underlying pathological mechanisms are needed.

## Figures and Tables

**Table 1:** Chloride-secretion-related genes in RNA-seq datasets of IBS-D colon.

Gene Name	Expression (Fold Change)	Adjusted *p*-Value
SLC12A2 (basolateral Na-K-Cl co-transporter, NKCC1)	−0.3 log2	0.883
CLCA1 (calcium-activated chloride channel regulator 1)	−0.6 log2	0.793
CFTR (cystic fibrosis transmembrane conductance regulator)	−0.3 log2	0.852

Sequencing data are deposited at the European Genome-Phenome Archive (EGA) database under record number EGAD50000000063.

**Table 2:** Gene expression of ENaC subunits in RNA-seq datasets of IBS-D colon.

Gene Name	Expression (Fold Change)	Adjusted *p*-Value
SCNN1A (α-ENaC)	1.2 log2	0.259
SCNN1B (β-ENaC)	1.3 log2	0.327
SCNN1G (γ-ENaC)	3.4 log2	0.240

The sequencing data are deposited at the European Genome-Phenome Archive (EGA).

**Table 3:** Expression of tight junction protein genes in RNA-seq datasets of IBS-D colon.

Gene Name	Expression (Fold Change)	Adjusted *p*-Value
CLDN1 (claudin-1)	−0.6 log2	0.785
CLDN3 (claudin-3)	0.8 log2	0.163
CLDN4 (claudin-4)	1.4 log2	0.006 *
CLDN5 (claudin-5)	−0.7 log2	0.601
CLDN8 (claudin-8)	0.7 log2	0.744
OCLN (occludin)	0.7 log2	0.438

* significant with *p* < 0.05. Sequencing data are deposited at the European Genome-Phenome Archive (EGA).

**Table 4:** Upstream regulator analysis. Top activated upstream regulators of immune function.

Upstream Regulator	*p*-Value of Overlap	Activation z-Score	Target Molecules
TNF-α	1.39∙10^−5^	3.370	ABTB2, ADM, ASS1, BDKRB1, CDKN1A, CLDN4, DUSP6, EHD1, KLF4, NOSTRIN
IL-1β	8.96∙10^−4^	2.427	ADM, ASS1, BDKRB1, CDKN1A, EHD1, ERRFI1, KLF4, PLB1, SLC25A25, SLC2A1
IFN-γ	2.45∙10^−3^	2.729	ABTB2, ADM, ASS1, CDKN1A, KLF4, MXD1, PDGFA, PLAAT4, PLAUR, PRKG2
LPS	2.62∙10^−3^	3.132	ADM, ASS1, BDKRB1, CDKN1A, EHD1, KCNK1, KLF4, MELTF, MXD1, PDGFA
CSF2	2.82∙10^−3^	2.111	CDKN1A, DUSP6, IFNLR1, PLAUR, SGK1, SLC2A1
IL-4	5.27∙10^−3^	2.215	ASS1, CDKN1A, IFNLR1, KCNK1, KLF4, MXD1, SLC25A25, SLC2A1, USP2

Footnote. Top upstream regulators of immune function, such as pro-inflammatory cytokines (IFN-γ, interferon gamma; TNF-α, tumor necrosis factor alpha; CSF2, colony stimulating factor; IL, interleukin), and effectors such as lipopolysaccharides (LPS) with significant activation of their downstream targets in colon specimens from patients with IBS-D (*n* = 4 patients and *n* = 4 healthy controls). Column 1: Gene name of the upstream regulator. Column 2: *p*-value of the overlap of the downstream target gene set and the pathway gene set. Column 3: Activation z-score. Column 4: Names of genes with expression changes. Sequencing data are deposited in the European Genome-Phenome Archive (EGA) database under record number EGAD50000000063. RNA-seq data were analyzed using Ingenuity Pathway Analysis (IPA) software (version 101138820). The complete list of genes differentially expressed in patients with IBS-D and controls, which are downstream targets of the upstream regulator, is provided in the supplement (Supplementary Table S1). The overlap *p*-value measures whether there is a statistically significant overlap between the dataset genes and the genes regulated by an upstream transcriptional regulator based on the literature and database values. It is calculated using Fisher’s exact test, and significance is generally attributed to *p*-values < 0.01. A statistical approach defines a quantity (z-score), which determines whether an upstream transcriptional regulator has significantly more “activated” predictions than “inhibited” predictions (z > 0) or vice versa (z < 0).

**Table 5:** Morphometric analysis of mucosal surface area and epithelial apoptotic rate in IBS-D.

	**Controls**	**IBS-D**
Mucosal surface area/serosal area	4.41 ± 0.71	5.16 ± 0.71
Apoptotic rate (percentage of TUNEL-positive cells)	1.0% ± 0.7%	0.8% ± 0.4%

The groups were not significantly different in Student’s *t*-test, n.s.

## Data Availability

Fastq files containing the raw unprocessed sequencing data and a raw data matrix table are deposited in the European Genome-Phenome Archive (EGA) database under record number EGAD50000000063, https://ega-archive.org/datasets/EGAD50000000063, accessed on 10 November 2023.

## Supplementary Materials

The following supporting information can be downloaded at: https://www.mdpi.com/article/10.3390/cells12242846/s1, Table S1: Upstream regulators in IBS-D. Entire list of activated, inactivated or affected pathways in upstream regulator analysis (Ingenuity IPA software, version 101138820, Qiagen Silicon Valley, Redwood, CA, USA).
